# Use of High‐Flow Nasal Cannula in Amyotrophic Lateral Sclerosis Patients Intolerant to Non‐Invasive Ventilation: A Case Series

**DOI:** 10.1002/rcr2.70744

**Published:** 2026-08-28

**Authors:** Fabio Varon‐Vega, Carlos Evelio Franco Parra, Doris Fabiola Adame Ochoa, Nancy Uricoechea, Eduardo Tuta‐Quintero, María Camila Martinez Ayala

**Affiliations:** ^1^ Critical Care Service Fundación Cardioinfantil‐Instituto de Cardiología Bogotá Colombia; ^2^ Critical Care and Lung Transplantation Service Fundación Neumológica Colombiana Bogotá Colombia; ^3^ Sistemas de terapia respiratoria, STR en Casa Bogotá Colombia; ^4^ Universidad Manuela Beltan Bogotá Colombia; ^5^ School of Medicine Universidad de la Sabana Chia Colombia

**Keywords:** amyotrophic lateral sclerosis, high‐flow nasal cannula, home care, non‐invasive ventilation, respiratory support

## Abstract

Non‐invasive ventilation (NIV) is standard respiratory support for amyotrophic lateral sclerosis (ALS), but intolerance may limit its use. High‐flow nasal cannula (HFNC) has emerged as a potential alternative in selected patients, but evidence regarding its use remains scarce. We describe 18 ALS patients' management with HFNC between 2014 and 2024 after documented intolerance to NIV. Mean age at HFNC initiation was 65.1 ± 12.5 years, and 66.7% of patients were female. Partial NIV intolerance occurred in 38.9% of cases, while 33.3% tolerated intermittent combined NIV and HFNC. During follow‐up, 50.0% of patients required hospitalization, 33.3% experienced respiratory infections, and 27.8% died. Arterial blood gas parameters remained stable, including PaCO_2_ (*p* = 0.314). Functional status declined significantly, with Barthel Index decreasing (35.0 ± 20.5 vs. 11.7 ± 11.3; *p* < 0.001), whereas respiratory muscle strength remained unchanged. HFNC was feasible as home‐based support in ALS patients intolerant to NIV, warranting prospective comparative evaluation.

## Graphical abstract

In this case series of 18 patients with amyotrophic lateral sclerosis and intolerance to non‐invasive ventilation, high‐flow nasal cannula was feasible as home‐based respiratory support. Gas exchange remained stable during follow‐up despite progressive functional decline, supporting further evaluation of HFNC as an alternative or complementary strategy in selected patients.

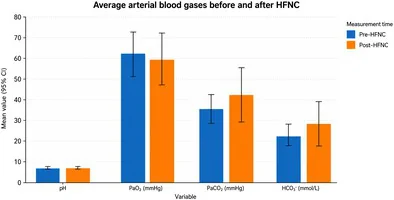

## 1 Introduction

Amyotrophic lateral sclerosis (ALS) is a progressive neurodegenerative disease characterized by degeneration of upper and lower motor neurons, resulting in progressive weakness of limb, bulbar, and respiratory muscles. Respiratory muscle involvement leads to dyspnea, sleep‐disordered breathing, chronic respiratory failure, and ultimately represents the leading cause of death in this population [1, 2, 3]. Non‐invasive ventilation (NIV) is the standard respiratory support for patients with ALS and has been shown to improve quality of life and prolong survival, with a median survival benefit of approximately 205 days, particularly among patients without severe bulbar dysfunction [4, 5, 6]. However, long‐term adherence to NIV may be compromised by poor tolerance related to interface discomfort, claustrophobia, excessive respiratory secretions, dysphagia, or difficulties maintaining an adequate mask seal [6, 7]. In patients who cannot tolerate NIV, invasive mechanical ventilation via tracheostomy may be considered, although this approach is associated with substantial ethical, clinical, and quality‐of‐life considerations [4, 5, 6, 7].

High‐flow nasal cannula (HFNC) delivers heated and humidified gas through a wide‐bore nasal interface at flow rates of up to 60 L/min with adjustable inspired oxygen fractions (FiO_2_). In addition to improving patient comfort, HFNC generates low levels of positive airway pressure, reduces anatomical dead space, and may decrease the work of breathing [8, 9]. Emerging evidence in patients with neuromuscular disorders suggests that HFNC may be better tolerated than NIV while providing adequate respiratory support in selected clinical scenarios. Nevertheless, evidence specifically addressing its use in patients with ALS remains scarce and is largely limited to small observational studies and case series [10, 11, 12]. Furthermore, most available evidence has been generated in tertiary care centers from high‐income countries at moderate altitudes, leaving important knowledge gaps regarding the use of HFNC in patients with ALS who are intolerant to NIV, particularly in high‐altitude environments and resource‐limited healthcare settings such as ours [10, 12, 13].

The objective of this study was to describe the sociodemographic, clinical, functional, and arterial blood gas characteristics of patients with ALS and documented intolerance to NIV who were managed with HFNC as an alternative home‐based respiratory support strategy. Additionally, we describe their clinical outcomes during follow‐up to provide real‐world evidence on the feasibility of HFNC in this challenging clinical setting.

## 2 Methods

### 2.1 Study Design

A retrospective, observational case series was conducted to characterize patients with ALS who were intolerant to NIV and subsequently managed with HFNC as an alternative respiratory support strategy.

### 2.2 Population and Sample

Patients followed between January 2014 and December 2024 in a specialized home care program for neuromuscular diseases were consecutively included using convenience sampling. Eligible patients had a confirmed clinical diagnosis of ALS, evidence of chronic respiratory failure, documented intolerance to NIV, and initiation of HFNC in the home setting. NIV intolerance was established from the medical records based on clinician documentation of persistent difficulties related to interface intolerance, claustrophobia, excessive respiratory secretions, dysphagia, behavioural intolerance, or anatomical factors precluding effective NIV use.

### 2.3 Procedures

Clinical information was obtained through a systematic review of electronic medical records. Variables collected included demographic characteristics, comorbidities, nutritional status, arterial blood gas measurements, respiratory muscle strength, ventilatory support characteristics, functional status, hospitalizations, respiratory infections, and mortality during follow‐up.

HFNC was delivered at home using heated humidified systems capable of providing flow rates of up to 60 L/min with adjustable inspired oxygen fraction (FiO_2_), titrated according to peripheral oxygen saturation (SpO_2_). Device selection, parameter adjustment, and longitudinal follow‐up were performed by a multidisciplinary home respiratory care team experienced in non‐invasive ventilatory support.

Functional status was assessed using the Cruz Roja Functional Classification, a two‐domain instrument (physical disability and mental capacity) graded from 0 (no impairment) to 5 (maximal impairment), with higher grades indicating greater disability (Table 1). Functional outcomes, including the Cruz Roja Functional Classification, Barthel Index, and respiratory muscle strength, were assessed at HFNC initiation (baseline) and at the last available follow‐up recorded in the electronic medical record. Because of the retrospective design, follow‐up duration was not standardized and varied according to each patient's clinical follow‐up within the home care program.

**TABLE 1:** Cruz Roja functional classification.

Grade	Physical disability	Mental capacity
0	Fully self‐sufficient; normal ambulation.	Completely normal.
1	Performs basic ADLs adequately; ambulates with some difficulty; normal continence.	Memory disturbance but normal conversation preserved.
2	Needs some assistance for ADLs; ambulates with cane/aid; normal or rare incontinence.	Memory ± orientation deficits; reasoned conversation possible but imperfect; behavioural changes; some self‐care difficulty; occasional incontinence.
3	Marked difficulty in many ADLs; ambulates only with assistance of ≥ 1 person; occasional incontinence.	Severe memory/orientation disturbance; unable to maintain coherent conversation; evident behavioural disorder; major self‐care limitations; frequent incontinence.
4	Requires help for almost all ADLs; ambulation only with assistance of ≥ 2 people; habitual incontinence.	Complete disorientation; established dementia; habitual incontinence.
5	Bed‐ or chair‐bound; requires continuous nursing care; total incontinence.	Advanced dementia; vegetative state with or without agitation; total incontinence.

### 2.4 Statistical Analysis

Categorical variables were summarized as absolute frequencies and percentages, whereas continuous variables were reported as mean ± standard deviation (SD) or median and interquartile range (IQR), according to their distribution. Normality was assessed using the Kolmogorov–Smirnov test. Comparisons between baseline and the last available follow‐up were performed using paired Student's *t*‐test or the Wilcoxon signed‐rank test, as appropriate. Exploratory comparisons between independent groups were performed using Student's *t*‐test or the Mann–Whitney *U* test for continuous variables and the chi‐square test or Fisher's exact test for categorical variables. Statistical analyses were performed using Microsoft Excel 2013.

## 3 Results

Eighteen patients diagnosed with ALS who received ventilatory support with HFNC after documented intolerance to NIV were analysed. The mean age at HFNC initiation was 65.1 years (SD: 12.5), with a mean age at diagnosis of 57.5 years (SD: 13.6). Females predominated, accounting for 66.7% (*n* = 12). The mean body mass index (BMI) was 23.6 kg/m^2^ (SD: 3.7). Regarding comorbidities, 22.2% (*n* = 4) had arterial hypertension, and 5.6% (*n* = 1) had diabetes mellitus. Obstructive sleep apnoea syndrome (OSAS) was recorded in 61.1% (*n* = 11) of cases. No history of neoplasms, thrombotic disease, or autoimmune disorders was identified (Table 2).

**TABLE 2:** Baseline characteristics of ALS patients managed with HFNC.

Patient/variable	Age	Age at diagnosis	Sex	BMI	Comorbidities
1	55	47	Female	24.5	OSAS
2	60	52	Female	21.3	
3	65	59	Female	23.8	Depression
4	83	71	Female	24.3	HTN, OSAS, COPD
5	58	45	Female	19.4	OSAS
6	76	67	Male	22.6	OSAS, RA
7	72	46	Male	18.5	
8	79	75	Male	27.6	COPD
9	43	33	Male	25.3	
10	52	45	Female	30.1	
11	78	70	Female	24.5	HTN
12	48	41	Female	23.4	
13	73	67	Male	23.7	DM
14	61	57	Male	17.3	HTN
15	59	57	Female	30.2	HTN
16	57	53	Female	17.8	
17	66	64	Female	24.4	
18	87	86	Female	26.2	

Abbreviations: COPD, chronic obstructive pulmonary disease; DM, diabetes mellitus; HTN, hypertension; OSAS, obstructive sleep apnea syndrome; RA, rheumatoid arthritis.

All patients included in this series had documented intolerance to NIV. In seven of the 18 cases (38.9%), intolerance was partial, characterized by difficulties adapting to the interface, feelings of claustrophobia, or discomfort with the delivered flow. Of the total, 6 patients (33.3%) tolerated short periods of NIV alternating with HFNC use. In these cases, the mean HFNC daily use duration was 4.67 h (SD: 0.82). During the observation period, 50% of patients required at least one hospitalization, and 33.3% experienced respiratory infections. Five patients (27.8%) died during follow‐up.

**FIGURE 1:**
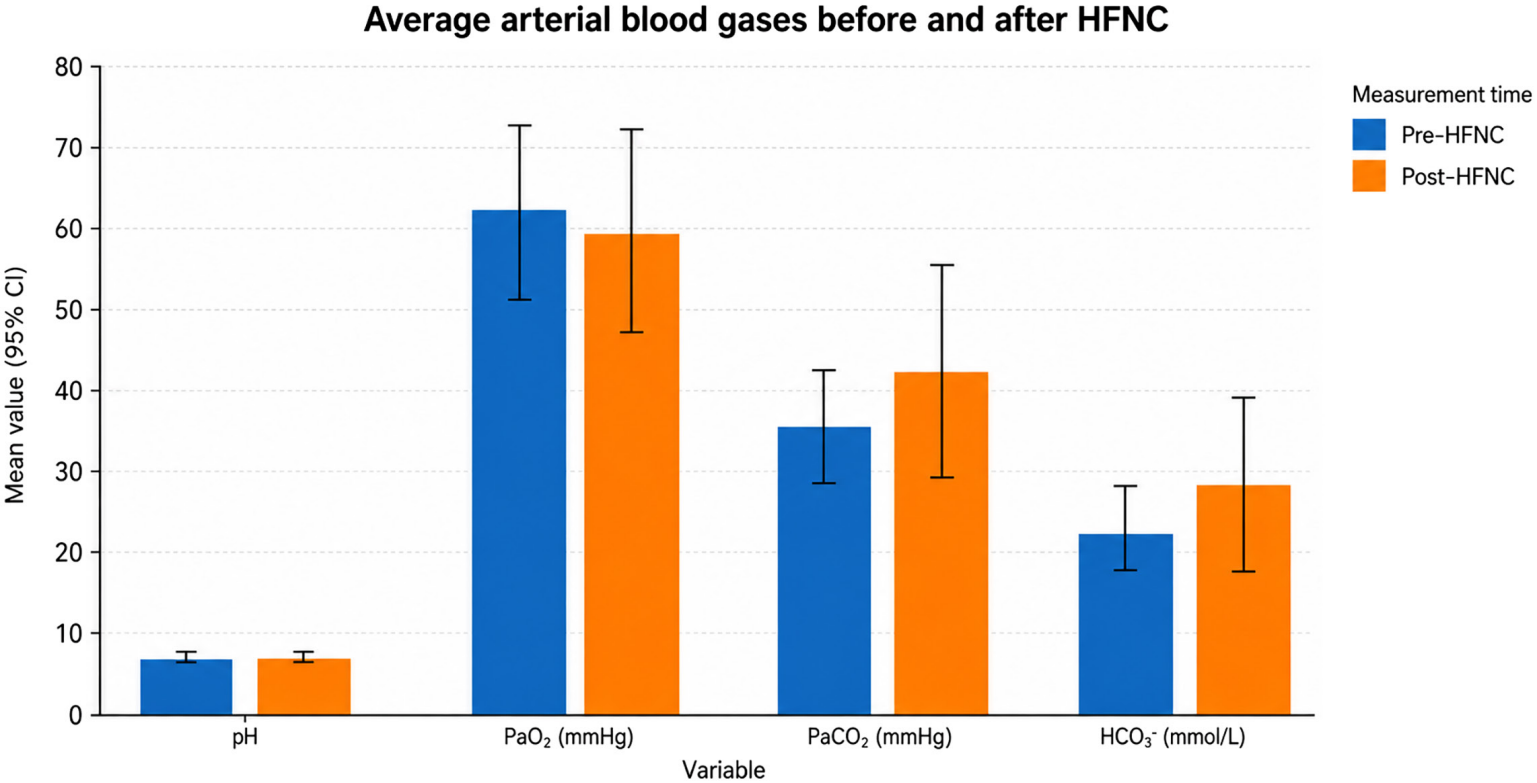
Average arterial blood gases before and after HFNC.

Arterial blood gases before and after HFNC initiation were compared. Baseline values showed a mean pH of 7.40 (SD: 0.05), PaO_2_ of 62.73 mmHg (SD: 6.95), PaCO_2_ of 36.35 mmHg (SD: 4.59), and bicarbonate (HCO_3_^−^) ranging from 16 to 29 mmol/L. After HFNC use, pH increased to 7.42 (SD: 0.02), PaO_2_ slightly decreased to 59.80 mmHg (SD: 8.17), PaCO_2_ increased to 42.40 mmHg (SD: 10.06), and HCO_3_^−^ rose to 27.80 mmol/L (SD: 9.26). None of these differences were statistically significant. Paired *t*‐test results showed a mean change in pH of −0.018 (95% CI: −0.093 to 0.057; *p* = 0.540), PaO_2_ of +2.12 mmHg (95% CI: −1.11 to 5.35; *p* = 0.143), PaCO_2_ of −5.66 mmHg (95% CI: −19.32 to 8.00; *p* = 0.314), and HCO_3_^−^ of −5.9 mmol/L (95% CI: −29.37 to 17.57; *p* = 0.482) (Figure 1).

During the last available follow‐up, a significant decline in functional status was observed. The Barthel Index decreased by a mean of 23.3 points, from 35.0 ± 20.5 at HFNC initiation to 11.7 ± 11.3 at the final assessment (*p* < 0.001), reflecting progressive loss of independence in activities of daily living, particularly walking and stair climbing. In contrast, respiratory muscle strength did not change significantly, with maximum inspiratory pressure (MIP) changing from 0.74 ± 0.44 to 0.70 ± 0.04 (*p* = 0.97) and maximum expiratory pressure (MEP) from 0.44 ± 0.24 to 0.46 ± 0.01 (*p* = 0.76).

Functional evolution according to the Cruz Roja Functional Classification also demonstrated progressive deterioration between HFNC initiation and the last available clinical assessment. At baseline, 77.8% of patients were classified as physical disability Grades 3 (38.9%) or 4 (38.9%), whereas 22.2% were Grade 5 (total dependence). At the final follow‐up evaluation, 55.6% had progressed to Grade 5, while only 5.6% remained at Grade 0. Likewise, the proportion of patients with mild mental impairment (Grade 1) increased from 22.2% at baseline to 38.9% at the last available follow‐up.

## 4 Discussion

This case series describes the clinical profile and outcomes of patients with ALS and documented intolerance to NIV who were managed with HFNC in a specialized home care program. Overall, HFNC was implemented in patients with advanced functional impairment and respiratory failure who had limited tolerance to conventional NIV. During follow‐up, arterial blood gas parameters remained stable without significant changes, whereas functional status progressively deteriorated, consistent with the natural history of ALS. Hospitalizations, respiratory infections, and deaths were frequent, reflecting the advanced stage of disease in this cohort rather than the ventilatory support strategy itself. These findings contribute real‐world evidence regarding the feasibility of HFNC as an alternative respiratory support option in selected patients who are unable to tolerate NIV [1, 2].

Our findings suggest that although HFNC did not significantly improve conventional arterial blood gas parameters, including oxygenation and carbon dioxide levels, its use was feasible in patients who were unable to tolerate NIV. These observations are consistent with those reported by Lionello et al. [10] who also described high patient acceptance of HFNC in individuals with neuromuscular diseases. The greater comfort associated with a nasal interface, preservation of oral communication and feeding, and reduced sensation of claustrophobia may contribute to the preference for HFNC over conventional NIV in selected patients [10].

Hospitalization and respiratory infection rates were comparable to those reported in other ALS cohorts [1, 14]. Unlike the study by Lionello et al. [10] in which three patients receiving continuous 24‐h HFNC experienced treatment failure, no complications directly attributable to HFNC were documented in our cohort. Although this finding should be interpreted cautiously because of the descriptive design and limited sample size, it may reflect the individualized adjustment of ventilatory support and close multidisciplinary follow‐up provided within the home care program.

Regarding gas exchange, HFNC was not associated with significant changes in arterial blood gas parameters, including PaCO_2_. This finding is consistent with the physiological mechanisms previously described. HFNC can improve secretion clearance, reduce anatomical dead space, and decrease the work of breathing; however, unlike NIV, it does not provide sufficient inspiratory pressure support to increase tidal volume or effectively correct alveolar hypoventilation and chronic hypercapnia [10, 11, 12]. Importantly, no evidence of respiratory acidosis or abrupt clinical deterioration attributable to CO_2_ retention was observed during follow‐up, suggesting that HFNC may maintain stable gas exchange in carefully selected patients despite its limited ventilatory assistance.

Functionally, patients exhibited a marked decline in independence for activities of daily living and increasing physical disability over time. This pattern is consistent with the expected progression of ALS rather than an effect of the respiratory support modality itself [14, 15]. In contrast, respiratory muscle strength, assessed by maximum inspiratory and expiratory pressures, remained relatively stable throughout follow‐up. This apparent dissociation suggests that overall functional deterioration in ALS is influenced not only by respiratory muscle weakness but also by progressive limb weakness, bulbar dysfunction and global neurological decline.

This study has several limitations. First, its retrospective design and the small sample size inherent to a case series limit the generalizability of the findings and preclude establishing causal relationships. Second, the absence of a comparison group prevented evaluation of the relative effectiveness of HFNC versus continued NIV or invasive ventilation. Third, follow‐up duration was not standardized because outcome assessments were based on the last available clinical evaluation recorded in the electronic medical record. The duration of HFNC therapy before the last follow‐up could not be consistently determined because treatment discontinuation dates were not systematically documented.

Functional outcomes, including the Barthel Index, Cruz Roja Functional Classification and respiratory muscle strength, were compared between HFNC initiation and the last available clinical assessment rather than at predefined follow‐up intervals, limiting direct comparisons of disease progression across patients. In addition, arterial blood gas measurements were obtained as part of routine clinical care rather than a standardized protocol. Consequently, we could not consistently determine whether baseline ABGs were obtained during NIV, the interval between HFNC initiation and follow‐up measurements, or the availability of serial ABGs for individual patients. Likewise, the duration of NIV before transition to HFNC was not systematically documented. These limitations restricted our ability to characterize the longitudinal physiological effects of HFNC and to evaluate whether previous NIV exposure influenced treatment tolerance or clinical outcomes.

HFNC was used as an alternative home‐based respiratory support strategy in patients with ALS and documented intolerance to NIV. Arterial blood gas parameters remained stable during follow‐up, while functional decline reflected the expected progression of the disease. These findings provide descriptive real‐world evidence on the use of HFNC in this challenging clinical setting and support the need for prospective comparative studies to better define its role in the respiratory management of patients with ALS.

## Funding

The authors have nothing to report.

## Ethics Statement

As a retrospective case series without experimental interventions, this study was classified as research without risk (‘investigación sin riesgo’) under Colombian regulations (Resolution 8430 of 1993, Ministry of Health), as it involved no intervention or intentional modification of patients' biological, physiological, psychological or social variables. Accordingly, approval by an ethics committee or Institutional Review Board (IRB) was not required. No additional sensitive data were collected, and no procedures outside standard clinical practice were performed. All clinical information was anonymized prior to analysis and publication. Written informed consent was obtained from all patients for the anonymized academic and scientific publication of their clinical information, including images. The study was conducted in accordance with the principles of beneficence, non‐maleficence, respect for persons and confidentiality.

## Conflicts of Interest

The authors declare no conflicts of interest.

## Data Availability

The data that support the findings of this study are available from the corresponding author upon reasonable request.
